# Surgery in a Patient with Haemophilia A and Lymphoma

**DOI:** 10.1155/2020/4852428

**Published:** 2020-03-18

**Authors:** M. S. Cruz, J. Santillan, J. Lesser, J. Ortiz, L. Forzani, P. Plaza

**Affiliations:** Fundación de la Hemofilia de Salta, Salta, Argentina

## Abstract

An increased incidence of haematologic malignancies and other cancer types among patients with haemophilia compared with matched controls has been reported in several longitudinal studies. Tumours initially misdiagnosed as haematomas and conversely haematomas mistaken for tumours have been reported. Here, we describe the case of a 43-year-old man with severe haemophilia A and a diffuse large B-cell lymphoma, originally diagnosed as a haematoma, who underwent a splenectomy and several associated surgeries as part of his lymphoma treatment. Perioperative treatment with octanate® (human coagulation factor VIII) enabled the successful performance of all surgical interventions required in the context of lymphoma treatment. Nevertheless, differential diagnosis of posttraumatic haematoma in patients with haemophilia should include the consideration of malignancy.

## 1. Introduction

Haemophilia A (HA) is an X-linked bleeding disorder that affects approximately 1 in 5000 male births [1]. It is characterised by spontaneous bleeding, mostly into muscles and joints. Severe bleeding episodes can be life-threatening [2], and repetitive bleeding into joints typically leads to irreversible joint damage and considerable morbidity (haemarthropathy) [3]. It is therefore important to prevent, or at least control, bleeding as effectively as possible. The standard of treatment for HA is administration of plasma-derived (pdFVIII) or recombinant coagulation factor VIII (rFVIII), either on-demand or prophylactically. For HA patients undergoing surgical procedures, maintaining adequate FVIII levels during and after the procedure is a crucial aspect of surgical management [2].

Here, we describe the case of a 43-year-old man with severe HA and a diffuse large B-cell lymphoma who underwent a splenectomy and several associated surgeries as part of his lymphoma treatment. All surgical procedures were successfully performed under FVIII cover.

## 2. Case Summary

A 43-year-old man with known HA presented with recurring back pain. He had been initially diagnosed with severe HA at 8 months of age and had received on-demand treatment with cryoprecipitate and transfusions until the age of 5 years and subsequently with pdFVIII. At age 6, he started intermittent prophylactic treatment using various pdFVIII concentrates at a dose of 25 international units (IU)/kg 3 times weekly. In the 1990s, the patient contracted hepatitis B and C as a result of treatment with a contaminated batch of Immunate®. For hepatitis C, a viral load of 2.3 × 10^6^ was determined, and the virus strain was characterised as genotype 1, subtype 1b.

Prophylactic treatment was stopped at age 22, and the patient returned to on-demand treatment with various pdFVIII concentrates, depending on availability and product price—mostly Hemofil®, Immunate®, octanate®, and Beriate®—until age 34. The patient experienced approximately four joint bleeds a year on this regimen and developed chronic haemarthrosis in his knees and right elbow as a consequence. Stomach ulcer that occurred during this time, at age 32, was successfully treated with pdFVIII on-demand (Hemofil® 30–50 IU/kg) and a red blood cell transfusion. In 2010, at age 34, contact was established with the Hemophilia Foundation of Salta, Argentina. At age 35, the patient started prophylactic treatment with octanate®, a pdFVIII concentrate stabilised with von Willebrand factor with proven low immunogenicity [4], 25 IU/kg 3 times weekly, and has experienced only two haemarthroses in the right knee in 8 years since then.

In January 2015, at age 38, the patient had a fall, and a splenic haematoma was detected by echography. He received 100 IU/kg of octanate® 3 times weekly for 3 months and was advised to rest. As a second echography in April 2015 showed that the haematoma had decreased in size, the dose was reduced to the usual 25 IU/kg 3 times weekly, and the patient resumed his normal activities, including travelling; however, he experienced recurring back pain. The octanate® dose was again increased to 100 IU/kg 3 times weekly for a month, after which treatment was resumed with the usual 25 IU/kg 3 times weekly.

A physical examination produced no noticeable findings apart from abdominal pain. An echography in August 2015 indicated a re-expansion of the splenic haematoma, and the octanate® dose was again increased to 100 IU/kg 3 times weekly. However, in March 2016, the haematoma enlarged further despite the higher octanate® dose, and the patient reported severe pain. As the imaging results still pointed towards a haematoma of the spleen, which had persisted for 15 months at this point, it was decided to opt for a splenectomy under octanate® cover as the patient was already receiving this product and had generally responded well to treatment. Tests performed in preparation for the surgery showed normal FVIII recovery and no inhibitors to FVIII.

The patient was referred to hospital on 27 March 2016; splenectomy was performed on 1 April 2016 under general anaesthesia. He received perioperative treatment with octanate® consisting of a presurgery loading dose of 37.5 IU/kg and postsurgical cover of 100 IU/kg/day for the first 3 days and 75 IU/kg/day for the next 3 days. Due to decreased haemoglobin levels after surgery, he received a transfusion of one unit of red blood cells.

A biopsy of the excised spleen was performed on 4 April 2016 and revealed a neoplasm that occupied a large part of the splenic parenchyma. Based on histological and immunohistochemical findings, a diagnosis of diffuse large B-cell lymphoma was made.

Invasive surgery was performed on 7 April 2016 to drain the abscess and the haematoma that had formed postsurgery and also because the presence of malign cells at the excision site was suspected. octanate® was administered as a presurgery loading dose of 100 IU/kg and as postoperative treatment (100 IU/kg/day for 2 days and 37.5 IU/kg twice a day for 10 days). The patient was discharged from hospital one week after laparoscopy; ambulatory checkups were done for a week after discharge. Following the postoperative treatment phase, the octanate® dose was reduced to 37.5 IU/kg 3 times weekly.

In addition to the surgery, chemotherapy was planned as part of the lymphoma treatment. In preparation of this, the patient received a 12-week course of oral sofosbuvir and daclatasvir in November 2016 and has been seronegative for hepatitis C since then. In parallel, he started treatment with entecavir for hepatitis B in December 2016 which was continued until December 2017.

In January 2017, a tumour recurrence in the splenectomy bed was detected by ultrasound and tomography. The patient immediately received 8 cycles of chemotherapy with rituximab, cyclophosphamide, doxorubicin, vincristine, and prednisone every three weeks (R-CHOP) between January and June 2017; aciclovir was given as concomitant treatment to prevent viral reactivation. Shrinkage of the abdominal mass was confirmed by computed tomography (CT). A positron emission tomography/CT in August 2017 showed a persisting abdominal mass with a diameter of 57 mm in the left flank, in contact with the abdominal wall. Standardised uptake values suggested infectious or inflammatory changes; however, persistent disease could not be excluded. An exploratory laparotomy for an abdominal wall biopsy was performed in November 2017 with octanate® cover (36 IU/kg before and after the procedure) due to incomplete wound healing from the surgery. Afterwards, the patient returned to prophylaxis at an octanate® dose of 25–30 IU/kg 3 times weekly. The result of the biopsy was negative.

The patient continued to undergo twice yearly checkups to detect possible relapses but did not receive any further lymphoma treatment. He continued prophylactic treatment with octanate®, and analysis using the Web-Accessible Population Pharmacokinetic Service (WAPPS-Hemo; http://www.wapps-hemo.org) and an octanate®-specific population PK model confirmed the appropriateness of his current prophylaxis schedule. Treatment with octanate®, both prophylactic and perioperative, was well-tolerated. A transient low-titre inhibitor (2.3 Bethesda units) was detected in January 2017; however, this was not clinically relevant as the titre had returned to undetectable levels 7 months later, without a change in therapy.

## 3. Discussion

**Figure 1:**
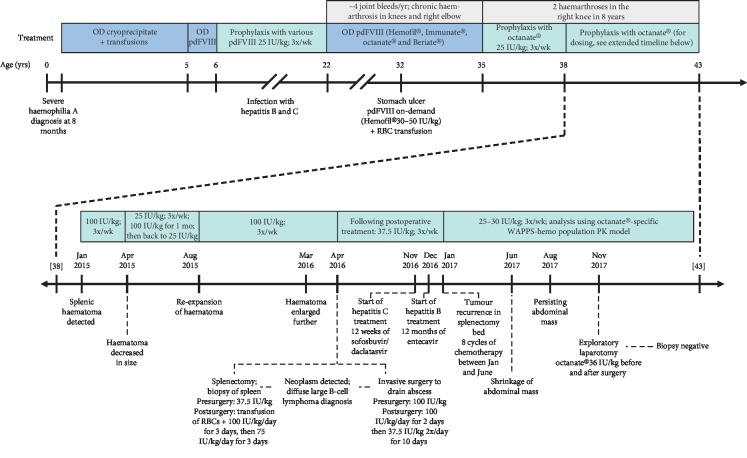
Timeline of key events.

In this 43-year-old patient with severe HA and a diffuse large B-cell lymphoma, perioperative treatment with octanate® has enabled the successful performance of all surgical interventions required in the context of lymphoma treatment (Figure 1).

A total octanate® dose of 762.5 IU/kg, including a loading dose of 37.5 IU/kg followed by 10 days of postsurgical treatment with a total dose of 725 IU/kg, was planned to cover the splenectomy (the total dose administered was only 562.5 IU/kg because the treatment schedule was interrupted for another surgery). Guidelines for the management of HA recommend preoperative doses of 50–80 IU/kg for minor surgery and 80–100 IU/kg for major surgery. Recommended postprocedure dose for minor surgery is 30–80 IU/kg on postoperative days 1–3 and 60–80, 40–60 and 30–50 IU/kg on postoperative days 1–3, 4–6, and 7–14, respectively, for major surgery [2]. The core SmPC for FVIII products by the European Medicines Agency suggests the use of 30–60 IU/kg every day for a least 1 day for minor surgery and 60–80 IU/kg every 8–24 hours for major surgery until wound healing [5]. Planned dose and duration of treatment for the splenectomy are also in line with surgical procedures (both major and minor) performed under octanate® cover during clinical studies in previously treated patients (PTPs), where the duration of treatment ranged from 1–20 days and the mean dose per exposure day (ED) from 28.6–68.3 IU/kg (Octapharma, data on file). For other pdFVIII products, similar data have been reported: loading doses ranged from 25–200 IU/kg, mean doses per ED were 50–70 IU/kg, and treatment for up to 10 days was common [6–9].

An increased incidence of haematologic malignancies and other cancer types among patients with haemophilia compared with matched controls has been reported in several longitudinal studies [10, 11]. Close follow-up for malignancies in these patients is therefore recommended [11]. In fact, several cases of haemophilia patients with tumours that were initially thought to be haematoma have been reported [12, 13]. Simple haematomas have also been mistaken for tumours [14–16]; nevertheless, differential diagnosis of posttraumatic haematoma in patients with haemophilia should include the consideration of malignancy [13].

In conclusion, this case report confirms the efficacy and tolerability of octanate® as surgical prophylaxis and provides further evidence showing that adequate FVIII cover enables major surgeries to be performed effectively in patients with severe HA.
